# Cultural Methods for the Control of the Invasive Japanese Stiltgrass (*Microstegium vimineum*) in Stream Restoration

**DOI:** 10.3390/plants15030477

**Published:** 2026-02-03

**Authors:** Robert A. Sullivan, Douglas A. DeBerry

**Affiliations:** 1 Department of Biology, College of William & Mary, Williamsburg, VA 23187, USA; 2 Environment & Sustainability Program, College of William & Mary, Williamsburg, VA 23187, USA

**Keywords:** stream restoration, cultural control, invasive species management, *Microstegium vimineum*, ecological restoration

## Abstract

*Microstegium vimineum* (Japanese stiltgrass) is one of the most invasive plant species in the eastern United States, posing a consistent problem to practitioners working in stream restoration and often necessitating treatment using non-selective herbicides to reduce invasion. Herbicide use frequently results in collateral damage to desirable native species and can lead to reinvasion after treatment. This study evaluated alternatives to herbicide referred to collectively as cultural controls, the use of which draws conceptually from the interaction of stress and disturbance in plant communities that predicts reduced invasion and increased competitive success of native species with higher levels of environmental stress. We tested several preventative cultural approaches, including (intended stressor in parentheses): (1) canopy shade (light limitation), (2) sawdust soil amendments (short-term nitrogen limitation), (3) wood mulch soil amendments (longer-term nitrogen limitation), and (4) double seeding rates (native species competition), as well as a combination of these treatments. Over a two-year field study within a restored stream corridor, we found that high carbon: nitrogen ratio soil amendments such as sawdust were the most effective at attenuating *M. vimineum* invasion and that shade promoted native species competition with this invader. Our results suggest a set of best practices that stream restoration practitioners could consider during the design and construction phases of a stream restoration project, particularly on sites with increased risk of *M. vimineum* incursion.

## 1. Introduction

**Figure 1:**
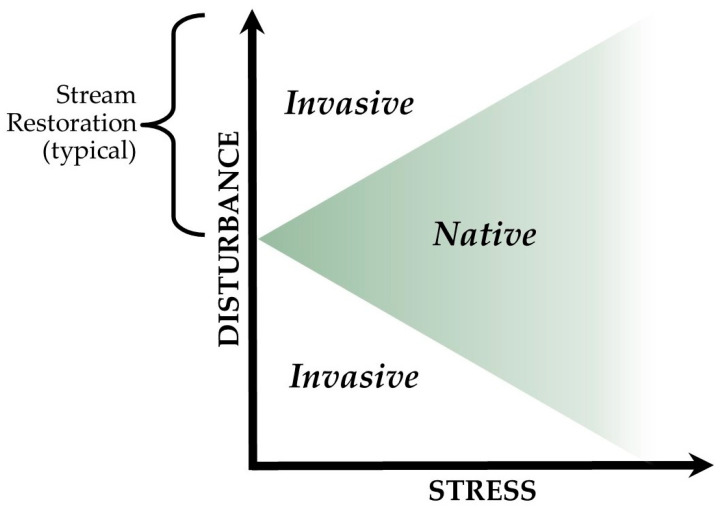
Relationship between stress, disturbance, plant invasion, and typical stream restoration scenarios (adapted from [4]).

Invasive plants are a constant issue for both our environment and our economies [1]. Invaders can cause deterioration of local ecosystems, with a staggering $190 billion being spent on treatment of invasive vegetation in the United States alone over the past several decades [2]. Invasive species are defined as those that enter an area that they previously did not inhabit and cause harm to the established ecosystem and/or human health [3,4]. Due to their deleterious effects, invasive plants are often treated with non-selective herbicides such as glyphosate. The issue with these types of herbicides is that they result in collateral damage to desirable native species in the invaded community [5,6,7]. This often leaves the area disturbed (i.e., removal of biomass), and in situations where habitats are also characterized by low levels of environmental stress (e.g., high nutrient and sunlight levels), sites are left susceptible to invasion [4] (see Figure 1). Removing invaders with herbicides often opens the treated area for reinvasion or incursion by new invaders and ultimately may augment the invasion instead of stopping or attenuating it [8].

### 1.1. Stream Restoration and Invasion

The above issues are especially problematic in ecological restoration, owing mostly to the fact that the practices used to create, restore, or enhance ecological conditions are often the same types of disturbances that leave a site vulnerable to invasion [9]. This is particularly true of stream restoration sites, which are characterized by open energy cycles and are exposed to multiple modes of plant dispersal from the watershed (e.g., flowing water and flooding in the riparian zone) [10] (see Figure 1). Invasive plant management on these sites has increased considerably in recent decades, and in most cases, it is compulsory, i.e., it is required as a condition of an environmental permit or a banking agreement for stream restoration after invasive plant species colonize [11]. This reactive posture to management typically results in use of non-selective herbicides and significant collateral damage to native species [6], but what is needed is a proactive approach to stream restoration with best practices aimed at inhibiting invasion from the start. Cultural invasive species control methods have the capacity to provide a means for such a proactive approach, but little field research has been completed to address the efficacy of these treatments in stream restoration.

### 1.2. Cultural Methods for Invasive Plant Control

As alternatives to herbicide, cultural treatments are land management approaches derived from agriculture, horticulture, and related disciplines to drive the outcomes of plant community composition toward desired goals [12]. Examples include use of non-chemical means such as prescribed burning, flooding or draining, livestock grazing, solarization, mulching, light manipulation, and strategic planting [13,14]. The latter three approaches—mulching, shade, and strategic planting—are of interest in stream restoration because they are scalable to the level of an entire restoration site and would not be restricted by issues like federal, state, or local ordinances or prohibitions (fire, flooding, draining); inability to target invaders (grazing); or impracticability (solarization).

Some invaders are inhibited by shade [9], highly competitive native plants [15], and nitrogen limitation [11]. Certain cultural treatments have the capacity to leverage these relationships and reduce the risk of invasion. Examples include planting or promoting natural tree canopies to increase shade [16], planting a high density of native species to increase competition with invaders [17,18], and using processed wood soil amendments to stimulate a nitrogen limitation [19,20,21]. The latter technique reduces available nitrogen by increasing the carbon:nitrogen (C:N) ratio in the soil, which increases microbial metabolism and causes uptake and immobilization of available nitrogen that could otherwise be used by plants [22]. These cultural treatments offer an alternative to the short-term gains but potential long-term losses of biodiversity and ecosystem health that can result from systematic use of herbicides.

### 1.3. Target Species: Microstegium vimineum

*Microstegium vimineum* (Trinius) A. Camus (Poaceae; Japanese stiltgrass) is one of the most common invaders on stream and wetland restoration sites in the United States [23]. *M. vimineum* is an annual grass introduced from East Asia that was first recorded in North America over 100 years ago and has since spread across the eastern U.S. [24,25]. It can grow to over a meter tall and forms dense monocultures when established [26]. *M. vimineum* also exhibits some level of shade tolerance [25,27], but recent research has suggested that relative amount of shade is important and could potentially be used to inhibit this species [9,11]. In addition to reproducing and spreading clonally by vegetatively rooting at nodes along its stolons, it is also a prolific seeder, has high seed viability, and forms persistent seed banks with seeds viable over three years [25,28,29,30], making it highly invasive and difficult to treat with chemical methods. Previous studies have confirmed that *M. vimineum* reduces native plant diversity [27,31] and can alter insect community structure [32]. It also has shallow roots [33], which can lead to severe erosion over time at restored stream sites [34], undoing the initial work to restore the streambanks and natural profile of a repaired or newly constructed channel. Importantly, flowing water is a dispersal mechanism for *M. vimineum* seeds, so floodwaters in unidirectional lotic systems contribute greatly to its expansion and distribution in watersheds. Much attention has been given to studying *M. vimineum* in these types of habitats [25,35,36], and its invasion potential on stream restoration sites is of particular importance in the Mid-Atlantic Region [37]. This combination of traits and factors makes *M. vimineum* an important target for strategic control methods in stream restoration.

### 1.4. Study Purpose

The purpose of this study was to test the efficacy of cultural methods as preventative management techniques for the control of the invasive *M. vimineum* in stream restoration. This was accomplished by designing and executing a large-scale field experiment on a stream restoration site in Reston, VA, USA. The study design included blocked treatments that evaluated shade, high C:N soil amendments using sawdust and wood mulch (mulch hereafter) as unique treatments, and high seeding density, all of which were implemented as individual trials and in different combinations to test potential interactions. The trials were staged in areas that were already dominated by *M. vimineum*, ensuring that the seedbank at the study sites contained the invader and all sites were at risk of re-invasion once the trials were executed. We hypothesized that both shade and wood amendments would be effective management techniques, with the more labile sawdust amendments providing short-term benefits and the more refractory mulch providing longer-term effects. We further hypothesized that high native seeding density by itself might not be effective but when combined with the other treatments would enhance control of *M. vimineum* due to positive effects of competition.

## 2. Methods

### 2.1. Study Site

**Figure 2:**
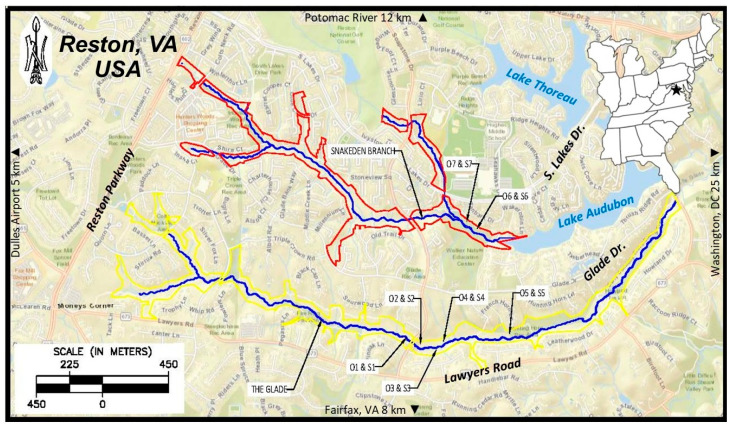
Study site, located within Reston, VA, USA, along the streams of Snakeden Branch (outlined in red) and The Glade (outlined in yellow). Block locations for shaded (S) and open (O) experimental conditions are shown [Inset map: star indicates location of project site in eastern USA].

The study site is located within the Northern Virginia Stream Restoration Bank (NVSRB), positioned in Reston, VA, USA (Figure 2). This site was selected because it is a restored stream system with areas in the restoration corridor dominated by *M. vimineum*, and also has a mix of open and closed canopies within those invaded areas allowing for evaluation of shading effects. The two main streams in the NVSRB corridor are The Glade and Snakeden Branch, both of which are part of the Difficult Run watershed, a tributary of the Potomac River. The restored sections of both streams collectively total over 17.5 km, with an emphasis on correcting stream reaches that had undergone extensive straightening and channelization since the development of the Reston area starting in the 1960s [38]. Both streams are low-order perennial channels supporting an average baseflow of between 0.01 and 0.03 cubic meters per second [39], and the soils within the riparian corridors are generally silt loam to loam in texture, grading to clay loam mixed with gravel at depth [40]. Most restoration activities were completed in 2008 and 2009 and involved increasing stream sinuosity, creating step-pool habitat, and leveling and stabilizing eroded streambanks to reduce future erosion and channelization within the watershed. The restoration process necessarily resulted in disturbance by removing the established biomass from the streambanks to fix the channel issues. Over the span of the next several years, the adjacent floodplain was subject to increases in the invader *M. vimineum*.

### 2.2. Block Selection

**Figure 3:**
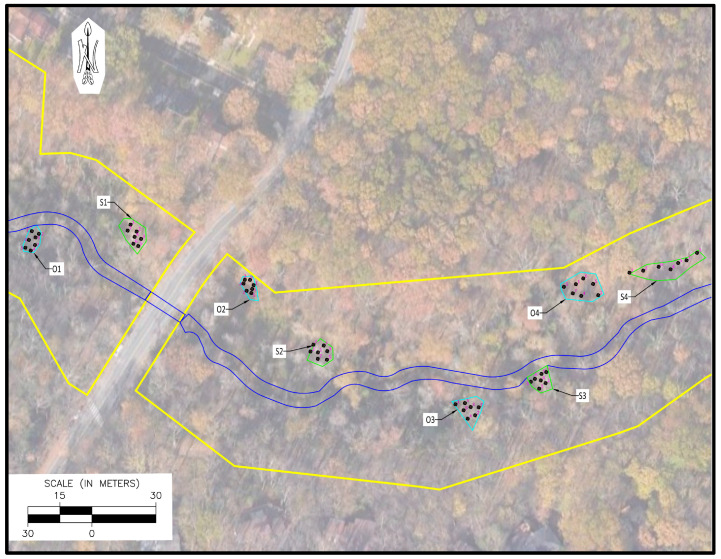
Example of open (blue) and shaded (green) experimental blocks—each with seven randomized plots shown—in a representative reach of the study corridor.

Areas overwhelmingly dominated by *M. vimineum* (>75% cover) within the study corridor were identified during site reconnaissance for experimental site suitability. Sites were initially screened to ensure similar geomorphic position (i.e., within the 100-year floodplain of the stream restoration corridor, which is the nearly level section of the floodplain on both sides of the channel) and that no selected sites were within areas previously designated as wetlands. After initial screening, the final selected experimental sites included “open” canopy areas and “shaded” canopy areas, which were defined as areas with <50% canopy cover and >75% canopy cover, respectively [11]. To verify shade conditions for each block type, canopy cover was estimated using a tripod-mounted Nikon D7100 digital single-lens reflex camera with a Sigma 8 mm f/3.5 circular fisheye lens. The tripod/camera setup was placed in the center of each block with the camera hand-leveled at 1.5 m above the ground surface to record a skyward photograph. Photos were post-processed using the Hemispherical 2.0 plugin to the photo-processing software ImageJ Version 1.54h, which converted the image to a binary map (i.e., sky converted to white pixels, canopy converted to black pixels; [41]). Canopy cover was then recorded as black pixel density (i.e., ratio of black pixels to all pixels). Once canopy cover was confirmed, study sites were delineated into an “open block” paired with a “shaded block”, with each block of sufficient size to allow for seven 1.5 m × 1.5 m treatment plots (Figure 3).

Prior to plot setup, soils were tested in all selected blocks to ensure similar baseline edaphic conditions. Soil samples were taken by coring 10 cm of soil from representative areas within each selected block using a 5 cm diameter soil corer, then submitted to the Virginia Tech Soil Testing Laboratory for analysis. At the lab, chemical variables were measured with Mehlich extractions for bulk nutrients, Elementar high-temperature combustion for total values of C and N, automated pH analyzer for pH values of wet samples at a 1:1 soil:water ratio, and normal mechanical analysis for soil texture [42].

The above site selection process resulted in 14 blocks across the study corridor (seven shade and seven open). Within each block, the seven 1.5 m × 1.5 m plots were randomly distributed throughout the block using the ArcGIS Pro 3.1 random point feature with a plot buffer of at least one meter.

### 2.3. Plot Setup: Experimental Trials

**Figure 4:**
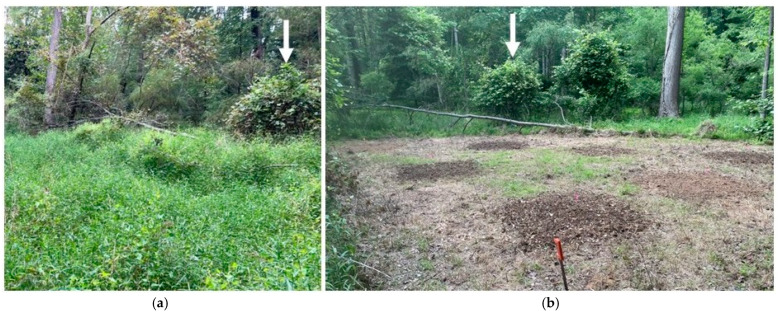
Representative block (O3) showing: (**a**) pre-experimental conditions with dominance of *M. vimineum* in foreground, and (**b**) plot setup after clearing, soil amendments, and seed application. Arrow shows the location of the same reference shrub in each image, with the restored stream channel located approximately 10 m farther into the floodplain beyond the indicated shrub.

In late spring 2023, blocks were mowed as close to ground level as possible. Immediately following mowing, a sub-meter GPS unit was used to wayfind to each of the 7 GIS-randomized plot locations within each block, and the 1.5 m × 1.5 m plots were centered on each point for a total of 98 plots over the 14 blocks (7 plots per block, 7 shade blocks, 7 open blocks; Figure 3 and Figure 4). Plot corners were marked, and each plot was flagged and numbered with a unique plot identifier.

Following the experimental stakeout, plots were raked clean of surface detritus then tilled using a hand-operated front-tine rototiller to simulate a newly constructed stream restoration site (i.e., soils reworked and rough-graded); then plots were treated with one of the management strategies in Table 1.

All soil amendment trials were re-tilled to mix the amended materials into the surface soil profile, then hand-raked to smooth the soil surface to final grade (Figure 4). The remaining non-amended plots were also hand-raked and smoothed at this time. Each plot was seeded by hand using a seeding rate equivalent to 67.2 kg/ha with the exception of the high-seed-density treatments, which were applied at double the rate (134.4 kg/ha). To ensure independence of experimental conditions in each block, plots were maintained throughout the growing season in 2023 and 2024 by mowing a 1 m buffer around each plot using contractor-grade weed trimmers.

### 2.4. Data Collection

At the end of peak growing season in 2023 and 2024 (e.g., early September), all plants within a 1 m^2^ sampling frame nested within each of the 98 experimental plots were identified to species level and quantified using the following abundance estimation procedures:

- Percent cover estimation using a cover class scale and taking the midpoints of the classes for analysis [44]. The cover classes, with midpoints in parentheses (rounded to the nearest whole integer), included: 0–1% (1%), 1–5% (3%), 5–25% (15%), 25–50% (38%), 50–75% (63%), 75–95% (85%), and 95–100% (98%).
- Density estimation using a logarithmic density class scale [45] in which the midpoint of the arithmetic interval represented by the logarithmic density class (log_2_N) is recorded for analysis. The density classes, with midpoints in parentheses, included: 1 (1), 2 (2), 3–5 (4), 6–11 (8), 12–23 (16), 24–47 (32), 48–95 (64), 96–191 (128), 192–383 (256), 384–767 (512), and 768–1535 (1024).
- Both cover class and density class were converted to relative cover and density and summed to produce an importance value (IV) [44], which ranged from 0.0 to 2.0. The IV for each species was used in statistical analysis of the community data in each plot.

From the center of each plot, canopy photos and soil samples were taken concurrently with the vegetation data collection using the methods described above.

### 2.5. Statistical Analysis

For statistical analysis, the ratio of *Microstegium* IV to the sum of all native species IV (MIVI:Natives) within each plot was treated as the primary response variable, with *M. vimineum* IV (MIVI) by itself also evaluated for linear correlations with environmental parameters. We used MIVI:Natives specifically to evaluate identity-level responses (e.g., invasive vs. native) to experimental treatments in the context of the overall vegetation community. For example, if two plots had the same invasive IV, but one had a higher overall IV of native species, the plot with the higher native dominance (i.e., lower invasive-to-native ratio) would be considered a more effective management scenario.

Various models (Table 2) were analyzed and compared using logistic regression to evaluate relative invasion under the experimental conditions. Bias-corrected Akaike’s Information Criterion (AICc) values were calculated to determine the most parsimonious model in each year of the two-year study. In all cases the response variable was log-transformed to correct for heteroscedasticity in the original dataset based on inspection of residuals vs. fitted values in the global model [46]. Linear relationships between measured environmental variables and *M. vimineum* response were then analyzed using the Spearman Rank-order Correlation and compared to AICc results for consistency (α = 0.05). The Spearman test was chosen due to its robustness to deviations from normality, as well as its ability to detect both linear and monotonic relationships, without appreciable loss of statistical power in comparison with parametric tests [47]. Finally, a nonmetric multidimensional scaling (NMDS) ordination of the abundance matrix was performed to evaluate underlying community structure across all plots in each year, with environmental variables fit to the final ordination model. The NMDS dissimilarity matrix was based on the Bray distance, which is a robust measure of community data similar to the type generated in this study [48]. All statistical tests were performed using R (Version 4.4.1).

## 3. Results

### 3.1. Floristic Composition and Dominance

In the first growing season of this experiment (2023), the overall dominance of *M. vimineum* was at 48.9% across the entire data set (i.e., average relative ratio of *M. vimineum* IV to all species IV within each plot) and ranged from 0.0% to 95.4% in individual plots. Of the native species found in our plots, the most common in the first year were *Dichanthelium clandestinum* (L.) Gould, *Bidens aristosa* (Michx.) Britton, and *Parathelypteris noveboracensis* (L.) Ching, along with a few species of unidentified sedges (*Carex* L. spp.) and grasses that were not in flower at the time of sampling. The overall native species richness of the data set from the first year was 58. Native richness in individual plots ranged from 9 to 27, with an overall mean of 19.2.

By the second year (2024), *M. vimineum* overall dominance increased to 69.0% across all plots and ranged from 11.9% to 96.9% in individual plots. The most common natives were *Carex lurida* Wahlenburg, *D. clandestinum*, *B. aristosa*, and *P. noveboracensis*, the first three of which had been included in the seed mix for the experiment. Overall native species richness was 60 and ranged from 12 to 27 in individual plots, with a mean of 20.5.

### 3.2. Initial Trial Analysis

**Figure 5:**
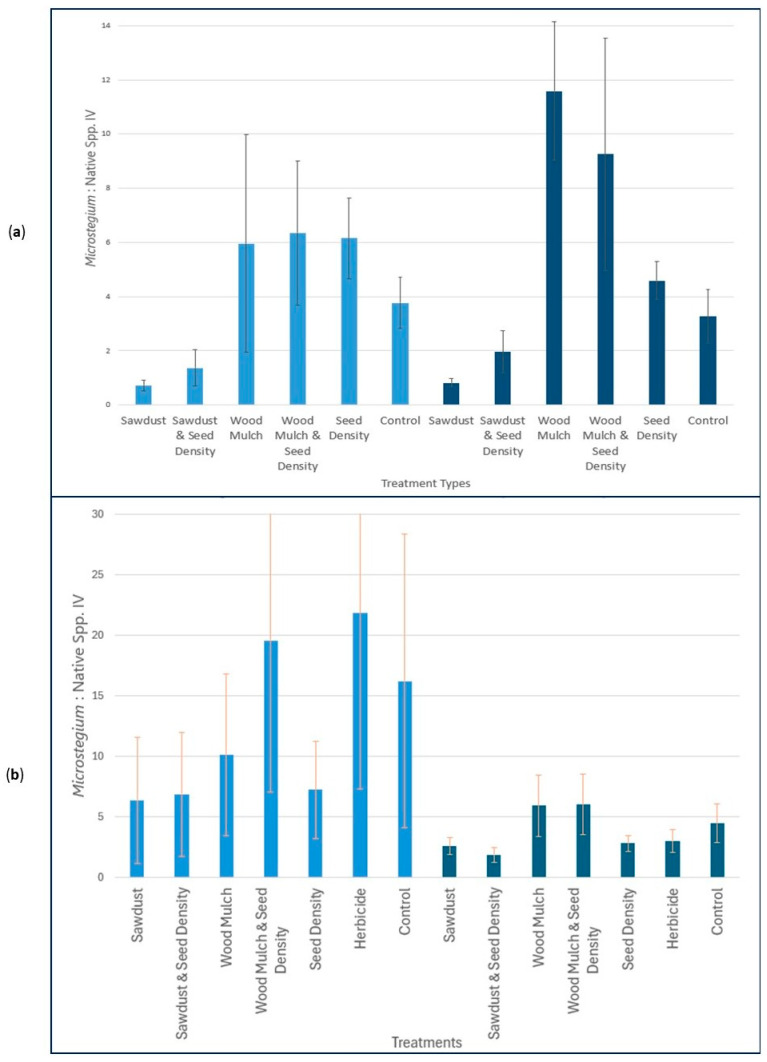
MIVI:Natives for each treatment. Light blue = open, dark blue = shade. Lines = standard error. (**a**) Year one results (2023). (**b**) Year two results (2024). (Note: Herbicide trials were not evaluated in year one because the treatment immediately preceded sampling, with 100% mortality in all herbicide-treated plots).

**Figure 6:**
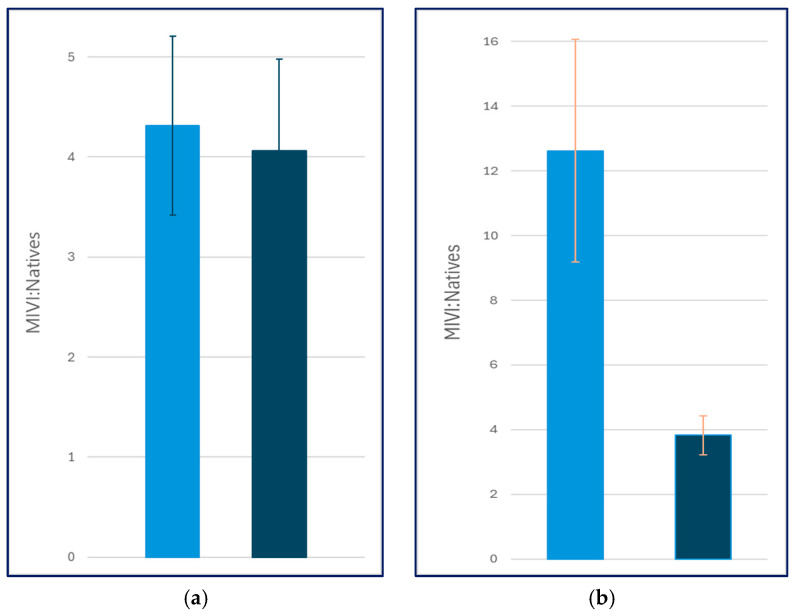
Overall MIVI:Natives between open (light blue) and shaded (dark blue) trials. (**a**) Year one results (2023), (**b**) Year two results (2024). Open and shade trials were significantly different (*p* = 0.01) by the end of the second growing season.

Graphical analysis of the data showed that sawdust treatments had the lowest overall MIVI:Natives values in the first growing season in both shaded and open trials, and the mulch treatments in the shaded plots had the highest MIVI:Natives ratios relative to all other trials (Figure 5a). Shaded plots had an overall lower average MIVI:Natives relative to open plots (Figure 6a), but the difference was not significant (*p* = 0.84) presumably due to the outsized effect of shaded mulch treatments. (Note: Herbicide trials were not analyzed in year one because herbicides had been applied prior to sampling, with 100% mortality in plots.)

By the end of the second growing season, the lowest MIVI:Natives values were recorded in the shaded sawdust trials, with the highest values in the open herbicide plots (Figure 5b). In addition, all shade trials showed lower MIVI:Natives compared to open canopy plots, a relationship that was significant (*p* = 0.01; Figure 6b).

### 3.3. Model Selection

Model selection results for the first year (2023) showed that the model best fitting the data was the sawdust treatment alone, with the shade + sawdust treatment as a plausible second model (ΔAICc = 2.53; Table 3). By the second growing season (2024), sawdust remained the most plausible model, with substantial support also for the trial combining shade + sawdust (ΔAICc = 1.07). The remaining models in the confidence set (i.e., cumulative Akaike weights up to 95%) were plausible but had considerably less support (i.e., ΔAICc > 3) compared to the best model.

### 3.4. Correlation and Ordination

The Spearman rank-order test for the first year showed that C:N was negatively correlated with MIVI, as were pH, calcium (Ca), and iron (Fe) (Table 4). Fe was also negatively correlated with MIVI:Natives. By the end of the second growing season, shade was negatively correlated with MIVI:Natives, and C:N was negatively correlated with MIVI. Among other soil variables, Fe was negatively correlated with both MIVI:Natives and MIVI, while pH was positively correlated with MIVI:Natives, and cation exchange capacity (CEC) was positively correlated with MIVI. Also, year two correlations showed a moderately positive relationship between soil N and MIVI, although the correlation was non-significant (*p* = 0.072, Table 4). Finally, although the interrelationships between environmental variables are not reported in Table 4, we note that C:N was negatively correlated with soil N in year two (rho = −0.379, *p* < 0.001) and Fe was positively correlated with phosphorus (P) in both year one (rho = 0.304, *p* = 0.012) and year two (rho = 0.359, *p* = 0.001).

**Figure 7:**
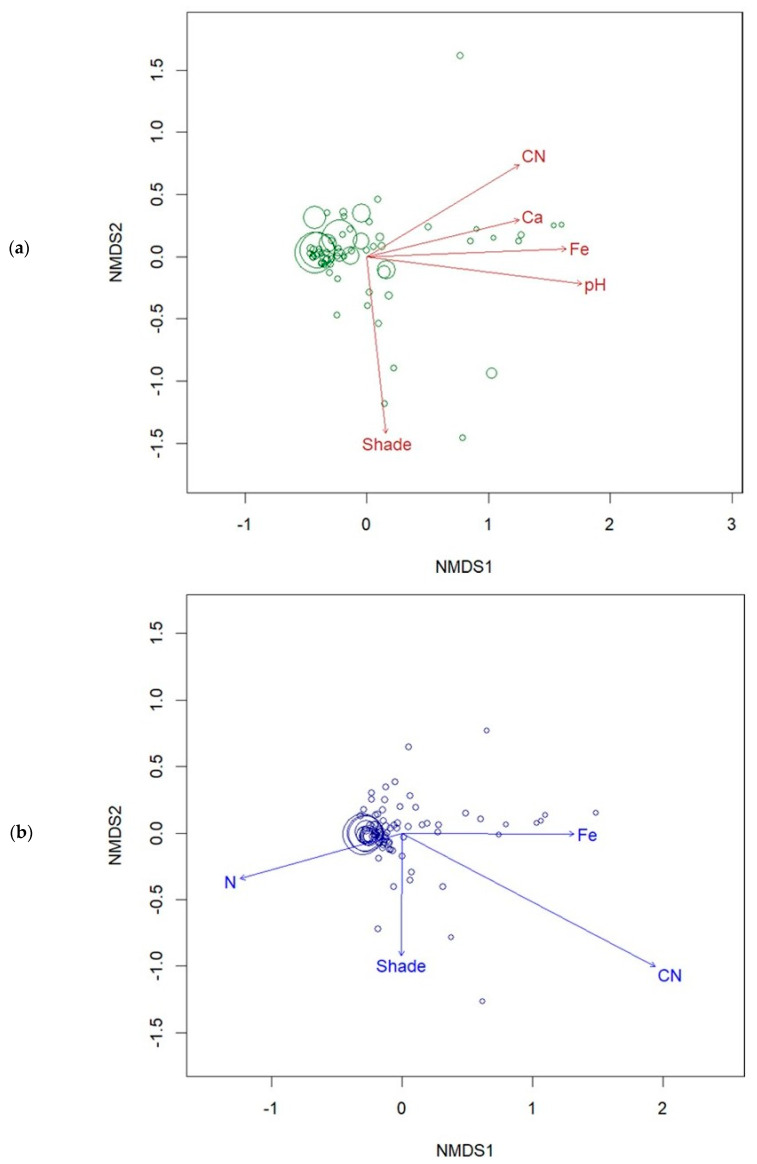
NMDS model of the relationship between plots (circles) and environmental variables (vectors). Larger circles represent plots with higher MIVI:Natives values, and vector length indicates strength of environmental correlation to the ordination. (**a**) Year one results (2023), (**b**) Year two results (2024).

The NMDS model for the first-year data supported the correlation analysis. The same environmental variables were significant in the ordination model, and all appear to be negatively related to MIVI:Natives (Figure 7a). One departure was shade: in the final NMDS solution, shade showed a significant relationship with the overall vegetation community and a clear negative relationship with MIVI:Natives.

In year two, the NMDS analysis identified shade, C:N, and Fe as important factors in structuring the community in our plots, and all were negatively related to MIVI:Natives. As shown in Figure 7b, C:N was the most important factor in the NDMS model as evidenced by vector length and was clearly negatively related to the response variable. Soil N was also a significant variable in the ordination and showed a positive relationship with MIVI:Natives. Like C:N, Fe was a significant variable in the model and showed a negative relationship with MIVI:Natives. Finally, shade emerged as a significant environmental factor in the analysis, and like C:N and Fe, it was negatively related to MIVI:Natives.

## 4. Discussion

This experiment—the first of its kind in a stream restoration context—was focused on preemptive control of the invader *M. vimineum*, particularly in situations where risk of future invasion is high. This is commonly the case in stream restoration undertaken in urbanized settings [11] but could also result from accidental introduction during construction regardless of site location or regional presence of the invader [49]. Our experimental setting was more aligned with the former scenario: an existing stream restoration corridor in an urbanized setting, portions of which were already invaded with *M. vimineum*, establishing a clear threat of invasion at the outset of the study. Further, our experimental design focused on blocked trials sited in areas where the baseline level of invasion was extensive, i.e., greater than 75% cover of *M. vimineum* in all blocks based on initial site screening. This ensured that all plots were exposed to a similar threat of reinvasion. Moreover, our trials were all initially prepared in the same manner to mimic rough and final grading in a stream restoration project. Thus, we feel confident that our results were the consequence of the trials themselves and not due to differential exposure to invasive propagules. The results were conspicuous: according to our data, a combination of high C:N wood amendments and shade could work to tip the scales in favor of more desirable native species on newly constructed stream restoration sites.

### 4.1. Soil Amendments

In our experiment, sawdust amendments increased C:N in the soil, which appeared to serve as a preemptive control for *M. vimineum* in the treated plots. This was borne out by the AICc analysis, which showed the sawdust trials as the best model fitting the data in both years of our experiment. This result was anticipated by [11] based on the outcomes of an observational study conducted on multiple invaded stream restoration sites throughout Virginia. Although we do not have data on microbial activity from our plots, the mechanism for *M. vimineum* control was most likely the bacterial immobilization of N creating an N-limiting condition in the soil [19]. The C:N ratio for sawdust treatments hovered around 20, which is the recorded level at which soil microbes typically start to immobilize nitrogen [50]. This N-limiting condition was supported by the negative correlation between C:N and N that was measured in our plots by the end of the second growing season (*p* < 0.001), and further undergirded by the year two NMDS model, which showed plots with increased N availability clearly supporting higher abundance of *M. vimineum* and vice versa (Figure 7b).

Unlike the sawdust amendments, mulch had the opposite intended effect, in most cases resulting in an increase in MIVI:Natives (see Figure 5a,b). The difference in effect could have been due to the higher surface-to-volume ratio of sawdust vs. mulch, which increases lability and makes it more easily broken down by microbes in the soil [51]. Our experiment was conducted over a two-year period, so given more time the mulch might have increased N-immobilization, but it is unclear whether it would have been as effective as sawdust. Regardless, the first few years post construction appear to be the most critical to establishing a competitive native herbaceous community and keeping the invaders out of a restoration site [52], so even if an eventual N-limitation could be established in the long term from the more refractory mulch amendments, we suspect that it might be too late for the desired outcome.

One surprising result was the increase in MIVI:Natives imposed by the mulch amendments in the first growing season regardless of the shade conditions (Figure 5a). Although it is not clear what drove this response, we noted anecdotally that it was much more difficult to mix the mulch materials into the soil and also achieve a similar elevation compared to the pre-amended soil in those plots, a scenario also encountered by others working with amended soils in wetland restoration [53]. This would have resulted in a more aerated soil environment, a condition that may have favored the shallow-rooted *M. vimineum* in comparison with our deeper-rooted native perennials in the seed mix [9].

### 4.2. Shade Treatment

Shade figured prominently in our model selection results, with the combined shade + sawdust treatment emerging as a viable model in both years. This was supported by the ordination results, which identified shade as an important environmental driver of the plant community throughout the experiment (Figure 7a,b). By the second growing season, shade also showed a significant negative linear relationship with the response variable (MIVI:Natives) based on the correlation analysis (rho = −0.317, *p* = 0.003), a finding that was evident in the graphical analysis of the response variable (Figure 5b and Figure 6b). This result supports the findings of DeBerry and Hunter [11], who recorded significant negative relationships between canopy cover and *M. vimineum* abundance on over 20 stream restoration sites invaded by this species throughout Virginia.

Although shade tolerance in *M. vimineum* is well documented, it is also a species that will aggressively exploit canopy openings and respond rapidly to available ground-level sunlight in forested floodplains [23,36], so it appears that the relative amount of light availability is important in forest understories [54]. The average canopy cover percentage measured in our shade trials was 79.9% and ranged from 68.2% to 89.5%. Based on our results, and those of DeBerry and Hunter [11], it seems that a target of 75% canopy cover or higher would create a more favorable understory shade condition for competitive control of *M. vimineum* via native herbaceous species. This, combined with using a labile high C:N substrate amendment like sawdust, could provide a very effective, proactive, and low-cost management alternative for *M. vimineum* on stream restoration sites, particularly in high-risk areas. The challenge for most stream restoration practitioners, however, is how to achieve a 75% canopy cover target within the active construction zone of a stream restoration corridor. Some suggestions are provided in Section 5 below.

### 4.3. Seeding Rate

The purpose behind applying an increased seeding rate was to stimulate early growth of native species and apply competitive pressure to reduce *M. vimineum* emergence from the seed bank [43]. In our high seeding rate plots, we observed the opposite effect, with an increase in MIVI:Natives and MIVI in those trials. In a similar experiment on *M. vimineum*, Flory [35] saw similar results with high native seeding rates reducing community productivity, hypothesizing that adding seeds attracted seed predators or increased soil pathogen activity and inhibited the existing native seed bank. Anecdotally, we observed birds foraging in plots after seeding, so we suspect that bird depredation was a possible reason for poor performance in our high seeding rate trials.

Our plots were seeded using a broadcast method where the seeds were evenly distributed over the surface of the soil, which is the normal seed application approach used by stream restoration practitioners (D. DeBerry, pers. obs.). Seed application could be improved by using a seed drill, which shallowly buries the seed, thus reducing risk of seed depredation, and also improves germination by increasing seed-to-soil contact [55]. Seed drilling might not be a viable option for some stream restoration sites due to the narrow corridor, difficult terrain, and challenges of shallow roots in existing forested areas, but where possible it is an advantageous alternative to broadcast seeding and may have improved the higher seeding rate outcomes in our experiment.

### 4.4. Herbicide vs. Cultural Methods

Use of a nonselective herbicide was effective at killing *M. vimineum* in plots but of course also killed all desirable native species. In the first year of the experiment, herbicide was applied during peak growing season, which is a recommended spray window for this invader in our region [56]. For this reason, effects were evaluated in the second year of the experiment after those trials had a full growing season to recover from the chemical application. By the end of the second year, herbicide plots in the open blocks showed the highest regrowth of *M. vimineum* observed in any plots across the entire two-year experiment (Figure 5b). This result was anticipated based on the work of others [8,57] and underscores the desirability of the cultural alternatives presented here.

### 4.5. Other Notable Observations

The negative correlation between Fe and both response variables in both years was conspicuous and is consistent with DeBerry and Hunter’s [11] observations on Fe, expected P-availability, and plant invasion in stream restoration sites. This can be explained by normal Fe-P dynamics in soils, where ferric iron (Fe (III)) immobilizes phosphate by precipitation [58,59]. Fe performs this role in its oxidized state, which was to be expected in the upland floodplains where our experiment was sited. This was the reason that we did not intentionally attempt to impose a P-limitation through soil amendments, because we expected that it would already be in limited supply in our trials. Others have proposed soil amendments for this purpose in wetland restoration to counteract the release of phosphate from Fe-complexes in the chemically reducing conditions of wetland soils [9], but we suspect it would not be as effective in the upland soils of stream floodplains.

Although we did not observe a direct relationship between P and invader presence (Table 4), the acid extraction protocols in our soil analysis would have measured both bioavailable P and Fe-complexed P [42,60]. Thus, Fe-immobilization of P in our soils would have been supported by a positive correlation between Fe and P, which we observed in both years (*p* = 0.012 and 0.001, respectively). In other words, acid-extractable P in our data represents the total amount in the soil, but Fe is a proxy for its lack of availability to plants in terrestrial soils, which is consistent with the prediction that nutrient stress limits invader abundance [11]. The fact that this relationship was not directly attributable to our management trials suggests that there was likely some variability in the soil oxidation state among the plots in our experiment.

## 5. Recommendations

Given the data presented here, stream restoration practitioners looking to preemptively control invaders like *M. vimineum* could incorporate soil amendments at the outset of a project to induce a stress response in the invader by reducing bioavailable N. In our study and others (e.g., [19]), the material recommended is processed wood with a consistency similar to sawdust. Stream restoration designers and managers could integrate sawdust at a volumetric ratio of 2:1 (soil:sawdust), which worked well in our experiment. Ideally, the amendments could be sourced from felled trees and shrubs directly onsite; however, if construction crews do not have a viable option for processing wood at that fine of a consistency, then it should be readily available and cheaply sourced from local sawmills. It is clear from our results that a high C:N ratio amendment can be effective at lowering bioavailable N and stimulating a nutrient limitation in the soil, especially in the first couple of years following construction of a stream restoration project. The effect on preemptive *M. vimineum* management was positive but did not appear to inhibit the native species in our experiment. One important caveat to note here is that soil arbuscular mycorrhizae fungi (AMF) have been shown to enhance nutrient uptake and growth in *M. vimineum* ([28], although see [54]); therefore, we recommend as a precautionary practice that soil amendments used for this purpose should not include deliberate AMF additions.

Restoration site managers should also focus on encouraging more shade in stream corridors. This can be performed by saving as many large canopy shade trees as possible and, where saving trees is not practical, by planting fast-growing trees from larger stock sizes (e.g., containerized trees rather than bare root or tubeling sizes). Fast-growing, broad-leaved trees are best suited for re-establishment of a canopy as quickly as possible to hasten canopy closure and decrease the risk of early invasion (see [11] for recommended species in our region).

We acknowledge that saving canopy trees in construction corridors is challenging and involves significant cost–benefit analysis due to the imposition of having to work around existing trees to re-grade bank slopes, reestablish functional floodplain elevations, or excavate new stream channels in restoration corridors [61]. However, saving trees should be a priority for stream restoration projects, and based on the work presented here and elsewhere (e.g., [11]) would be one of the most effective ways to keep invaders out, particularly in areas with high risk of invasion.

Examples of effective tree save construction strategies for stream restoration projects include: (1) prior to construction, conduct a detailed tree inventory within the limits-of-disturbance (LOD) using ground survey techniques with high location accuracy, noting tree species, size, and overall health, and marking trees in good to excellent condition that can be saved; (2) work with the design team early in the planning process to determine project constructability within tree protection zones (TPZs) for earmarked trees; (3) conduct a pre-construction walkthrough of the project corridor with a tree care expert (e.g., certified arborist) to strategize construction-phase tree save techniques and to identify critical root zones (CRZs); (4) reduce soil compaction and root zone impacts through use of equipment matting, temporary bridging, or wood mulch and/or with track-mounted or similar equipment designed to limit ground pressure; (5) use tree protection fencing, and armor trees in the LOD with fabric cladding or timbers to minimize physical damage from accidental collisions; (6) where allowable, use erosion and sedimentation control techniques such as trenchless silt fencing that will not impact the CRZ of any earmarked trees; and (7) carefully prune branches and/or use tree growth regulators (TGRs) to reduce impacts on trees in major construction zones [62,63,64,65,66,67].

Finally, although the high-seed-rate trials were not effective in our study, we believe that our results would have improved if we had used a method similar to seed drilling rather than just broadcasting the seed on top of the soil. Seed drilling should be used in any native seeding project where possible. Further, we suggest that a native seed mix focused on species that are highly competitive with invaders like *M. vimineum* will produce better results for preemptive control of invaders. Examples of native dominants from our seed mix that would work well in the Mid-Atlantic Region include *D. clandestinum*, *B. aristosa*, and *C. lurida*. Although we did not include a targeted cover crop in our mixes, aggressive but short-lived species could also work to inhibit invaders through competition, and more research on the use of cover crops in stream restoration is warranted.

## Figures and Tables

**Table 1:** Seven experimental trials replicated within each block. Individual trials were established within 1.5 m × 1.5 m plots randomly located within each block.

Short Name	Treatment Description
Sawdust	Induced nitrogen limitation using sawdust mixed into the top 10 cm of the soil profile at a 2:1 soil:sawdust volumetric ratio [19]
Mulch	Induced nitrogen limitation using processed wood mulch mixed into the top 10 cm of the soil profile at a 2:1 soil:mulch volumetric ratio
Seed Density	High seeding density using a diverse mix of 20 native herbaceous species for increased native competition [43]
Sawdust + Seed Density	Combination of sawdust and seed density treatments
Mulch + Seed Density	Combination of mulch and seed density treatments
Herbicide	Herbicide treatment using contractor-grade (18%) glyphosate mixed and applied in accordance with the label specifications (0.365 g per 1 L of water)
Control	No treatment

**Table 2:** List of all trials evaluated using AICc [trials were modeled based on the general formula log(Y) = β0 + β1 × (trial)].

List of Models Evaluated Based on Trials
Shade
Sawdust
Mulch
Seed Density
Shade + Sawdust
Shade + Mulch
Shade + Seed Density
Shade + Sawdust + Seed Density
Shade + Mulch + Seed Density
Herbicide
Control (no treatment)

**Table 3:** AICc results for year one (2023) and year two (2024). Only the models with highest level of support are shown, i.e., confidence set of models up to 95% based on Cum. Wt. (=cumulative Akaike weights).

2023 Models	K	AICc	ΔAICc	Cum. Wt.	2024 Models	K	AICc	ΔAICc	Cum. Wt.
sawdust	3	148.43	0	0.78	sawdust	3	231.85	0	0.46
shade + sawdust	5	150.96	2.53	1	shade + sawdust	5	232.92	1.07	0.72
					shade	3	235.34	3.49	0.8
					mulch	3	235.51	3.66	0.88
					shade + mulch	5	236.82	4.97	0.91
					control	3	237.33	5.48	0.94

**Table 4:** Results of Spearman rank-order correlation tests for year one (left columns) and year two (right columns) with only the environmental-to-response variable correlations shown. Significant correlations are highlighted in green for year one and blue for year two (*p* ≤ 0.05).

	Year One MIVI:Natives		Year One MIVI		Year Two MIVI:Natives		Year Two MIVI	
Variable	rho	*p*-Value	rho	*p*-Value	rho	*p*-Value	rho	*p*-Value
Shade	0.012	0.922	−0.012	0.925	−0.317	0.003	−0.069	0.532
C:N	−0.104	0.400	−0.399	0.001	−0.142	0.197	−0.431	<0.001
N	−0.129	0.299	−0.046	0.709	0.022	0.840	0.197	0.072
P	−0.088	0.478	−0.143	0.250	−0.030	0.784	−0.175	0.111
K	−0.137	0.268	−0.115	0.352	−0.123	0.266	0.049	0.661
Mn	−0.022	0.857	0.122	0.326	0.126	0.255	0.089	0.423
pH	−0.062	0.616	−0.468	<0.001	0.220	0.044	−0.154	0.162
Ca	−0.125	0.312	−0.364	0.002	0.128	0.247	0.004	0.972
Fe	−0.344	0.004	−0.542	<0.001	−0.251	0.021	−0.266	0.014
CEC	−0.115	0.354	−0.060	0.628	0.223	0.041	0.086	0.434

## Data Availability

The datasets generated during and/or analyzed during the current study are available from the authors upon request.
